# Supplementary material for: Patterns of Intron Gain and Loss in Fungi
Source: PLoS Biol. 2004 Nov 30;2(12):e422. doi: 10.1371/journal.pbio.0020422 (PMC532390; doi:10.1371/journal.pbio.0020422)
Supplement: Table S1 — Also available at http://genes.mit.edu/NielsenEtAl/. (4.3 MB ZIP). [file pbio.0020422.st001.zip › NielsenEtAl/html/1245.html]

AN2496.1.NCU00675.1.MG02781.1.FG08819.1


```
 CLUSTAL W (1.82) Multiple Sequence Alignments - Introns Inserted


Sequence 1: NCU00675.1	1125 aa
Sequence 2: MG02781.1	1175 aa
Sequence 3: FG08819.1	1117 aa
Sequence 4: AN2496.1	1156 aa
Alignment Length: 1234 aa
Number Identitical Residues: 260 aa
Alignment Score (without introns) 15176


MG02781.1 	--MNAIQQKCRPKHQVLVLKCYPRTTKGAVDVKPNSSELSYLLYYAASRKSKFQKVGSFL
NCU00675.1	--MNALQQKCRPKHQVLVLKCYPRTIKGAVDVKPNSSELSYLLFYCQSRRAKIQKVGSFL
FG08819.1 	--MNALQQKCRPKHQVLVLKCYPRTAKGAVDVKPNSSELSYLLYYATSRRSKIQKIGAFL
AN2496.1  	MPMETVRQTCRPKHQILVLKCYPQYQKGVQEVKPNSSELSYLLYYVSTRRSKLPKVSAFL
          	 .*::::*.******:*******:  **. :************:*  :*::*: *:.:**

MG02781.1 	EKKTASDVWRLRIG2NVQVTLQILEALIEKNPKDLPLFAPSVLKILDLVLKSNDITMVES
NCU00675.1	EKKTASDVYHQRIG2NVQVTLQILAALIEKSPKDLPLFASCVLSILEQVLKSSDITMVES
FG08819.1 	EKKTASDVWRLRIG2NVQVTLQILSALMEKLHKDSVLIAPFVLKILDTVLRSDDITMIES
AN2496.1  	EKRAARDVWRRKIG2NVQVTLQILSALIEKVPRDLPIFARSVLTIIETVLRSRDISMVED
          	**::* **:: :** ********* **:**  :*  ::*  **.*:: **:* **:*:*.

MG02781.1 	STPTFEAFCANHDASSLFADQAYLKQYESIVRQYASLASTRRSPGKTTPSKPVAMRWRNT
NCU00675.1	SIPTFQAFCENHDPTSLAADQAYFRQYVSVVQQYASLASTRPAPGKAQHSKPIALRWRNA
FG08819.1 	SLPTFGAFCDYHDAAFLLADQTYLRQYEEIVRLYAQLAST-AAPGRESLTTPVKVRWRNA
AN2496.1  	SIATFETFCRHQDMAALSAEQDFANQYRDVIQIYAGFAHEEQQHPSKISSLPQTIRWKNA
          	* .** :**  :* : * *:* : .** .::: ** :*           : *  :**:*:

MG02781.1 	GLEAIR-SVASSDALASVQGTQYDILVPMILENLWTDNEEFLEILLHRAHLEEKVDTDKL
NCU00675.1	GLEAIR-SVASSDALSSMVARQYDILVPMILENLWTENEDFLDVLLQRVQGDNNVE-DAP
FG08819.1 	GLEAIR-SVSTADALSSITGSQMHVIMPRILENLWSDTPDFLEILHQRLEEEEKVD-TEK
AN2496.1  	GLRAIKGAVSSEAGLAADGGDLLRIILPVIMENLYNGEDSFIESLEHKLHEAERNVPDPA
          	**.**:.:*::  .*::  .    :::* *:***:.   .*:: * :: .  :.  .   

MG02781.1 	LLRRRTSVATVRTAE---GGNEPNPIALSGSAMDVDKLAEEDIGVLAITCLKQIFVG-PN
NCU00675.1	LLRRRTSNATAQPSETTGGEPGPNPIAFLGTAVDVDKLAEEDIGVLAMQCLRQVFVA-PS
FG08819.1 	QLRRRTSIATVGTDG----ANDPNPVALSGTAGDVDKLAEEEVGVLAMQCLKSIFIV-PT
AN2496.1  	SRRRYSAVTVQTVDT-----AEGDPALAAQNIADVDRKAELDMRLLALRCLDQVIVNGSS
          	  ** :: :.             :*     .  ***: ** :: :**: ** .::: ...

MG02781.1 	RSQIHAATVALLKFIEERVNQDENVVKKSARTGRDSGWAIKIFGLISRWAPVQERYVILM
NCU00675.1	RSQTHNPTIALLRFIEERVDQNEQVVKRDAH-GKDNGWAIKVFLMAARWAPVADRFTILL
FG08819.1 	RAQIHGATVSLLKFIQEKVAQGNSVVELHDDRERDNGWAISIYGIIARWAPVQDRYTILV
AN2496.1  	RGQIRLTTRLVLDFILRKGQSNNTYLSLDTD---ENNWATSLIEVVAKWCPVQARFIISS
          	*.* : .*  :* ** .:  ..:  :.       :..** .:  : ::*.**  *: *  

MG02781.1 	TTMDTLVRMPLSDESMRHHIVWMAMMGALLRSDVNLIGLSVMDVLLALMQHMKRLVQLPG
NCU00675.1	TAIEVLTQRPLTDDNLRHHNTQAAMISALLRSDVNLIGLSVMDVLLNLLRHMQRLVQMPG
FG08819.1 	AALETLLRIPAQDATLDEQLALVTIMSSLLRSDVNLIGLSMMDVLLGLIKQMRKLFRLRS
AN2496.1  	AAMEVLFEIPPKEDTLDEAFTVIYIIDYLLKSSVNMIGLSVIDVLLGLMRYMAMLVSPAS
          	::::.* . *  : .: .  .   ::. **:*.**:****::**** *:: *  *.   .

MG02781.1 	DPSGASASDMLLPGQPDPRSPTTIAASTAAQATAAARKELLERIQQTIGDLATHVYYADQ
NCU00675.1	DPDSMRVEDETIG---------------TDEGAIGQRRELLFRLQQCVGDLATHVYYADQ
FG08819.1 	PASRSDDGNVATA--------------ESDTAVRQKNKHLVGRLELCIGDLATHVYYADQ
AN2496.1  	AKGTDEQPDSSEK-----------PGYDDGTNLSQKKKDLLVLLQKCIGDLTTHIYYGDQ
          	  .     :               ..          .:.*:  ::  :***:**:**.**

MG02781.1 	ISDMISAIIQRLRPSKSNSPGNTSPQGEKIDG-------QVASPEDSTVESLFSLTVAKV
NCU00675.1	ISDMIQTILLKLRPSRPTSPPNSSPNGERSD---------NGAAEDQPLESLFALTVAKI
FG08819.1 	IADMVSAIILRLKPSRASS-INSSPGGEKNGNEAGPGASTVELSESQQFDHYFCISTGRA
AN2496.1  	VVDMLRAILTRIKPPHGQDQVSAVIPEQLDG------------HLSEANPTSFFSTSAKV
          	: **: :*: :::*.:  .  .:    :  .              ..     *  : .: 

MG02781.1 	AALKAIKTILLVANPRTKMAG-NLGLARNKVSISVWEGTQWLLRDPEGLVRKAYADAVIT
NCU00675.1	AALKAIKAILWVASPRTKMSGGHINLSRNRVPIQTWDGTQWLLRDPDGLVRKAYVDAVVT
FG08819.1 	SGLRAIKEILLVANPQKKLTG-NLAMSRNPVPIHVWEGTHWLLRDPDGHVRKAYMEAIVT
AN2496.1  	CALRAIKNILLVANSQRPMTS-AGVESRNPVGIHVWEGTHWLLQDPQKEVRYAYVDALLY
          	..*:*** ** **..:  ::.     :** * * .*:**:***:**:  ** ** :*:: 

MG02781.1 	WLDRETVKGDLKAVDESAPNPRASIRYTRDLGKMDVSPARRAASSASTRREPKP--TRLH
NCU00675.1	WLDRETTPADSLARDESARTT------LKNRAAQENNLARRVVSSASARVDKSANAPRSH
FG08819.1 	WLDRETSFASEIAVEEKLPRSRSS---IKMSKEASSGINRRAVSNASHR-ERGSKPRPSQ
AN2496.1  	WLKLETNKNDLKLKDRSTTTALIS--ARRDFSNTSERAAKRATG--THHREKALVVAQSN
          	**. **   .    :..   .  :    :     .    :*...  : : :        :

MG02781.1 	FLNLIHVAIYDNALQ2---------------------LGINAVRYGLPMIFRLQEDIQEV
NCU00675.1	FLQLLHLAIYDHALQ~FVDYENDLVLLHVLLAKLVSQLGVNAAKFGIPMIFRLQEDIQDV
FG08819.1 	FLALLHLVIYDNALQ~YVDFETDLVLLHILLTRLVLQLGVNAARYGLPMIYRLQEDVQEI
AN2496.1  	FLRLLHLTIYDVALQ~SSTQEKEIRILHLLLASLVENLGINAARFGLPMILKLQDDMTTL
          	** *:*:.*** ***     ...        :    .**:**.::*:*** :**:*:  :

MG02781.1 	DAP---IGKVRVGCLVHGYLWALTEKFDFENTLVGRAIHNEIIRRRSKHFWIEGVHVPPP
NCU00675.1	ETP---LGKVRIGSLVHGYLWTLTEKFDCEGTAPGSAIHGEIIRRRSKNFWVEGINIPAP
FG08819.1 	ETP---LHKVRIAALCHGYFWALSEKFDFKESDIGQAIESEVARRKRKAFWVQGINMPPP
AN2496.1  	ENSNTQAAKVNIGSLVHGYLWVLSETFGLDTHRAGQEIYTEIEKRKSRSIWLDSIGFSLE
          	: ..:.  **.:..* ***:*.*:*.*. .    *  *  *: :*: : :*::.: ..  

MG02781.1 	LLELIGTPGMVKPQPKMPLNQIESEALLPFDD-RFALIDSICTGYQESFASPPTSPAASP
NCU00675.1	AVDLVGTPGQARPPPQMNMRDLESEALLPFDE-RASLVDCICTGYQELATSPPTSPTTSP
FG08819.1 	SLDQVGIPGEALPQPDWDSASLEREELLPFDD-REALVESVVARYHESLQAPPGSPGS-P
AN2496.1  	SFESIIKD----DRHALSHNTRETDKMTLFKDGVEEFVRRIEESYNR--TVPAHDPPTSP
          	 .: :                 * : :  *.:.   ::  :   *:.    *. .* ::*

MG02781.1 	GRSFTSPILGSQMSGLSPHTATDDEREVPSRFRDQMYVEWTRDLVIAMVQAGS-KSASLN
NCU00675.1	GRNFTHPMLG---STLSATPKDETQREVPAQFRELMLGDWTREAVLANTQAGSSQTASLN
FG08819.1 	GRNPSGPILG---SSLTSTGQADSDVELPVVFREHMLGDWSRDAAAAMLAAAG-KSESLT
AN2496.1  	GRNLGKPVIG----GYLPPANQQLSDLLPPVVREQMLSPWSKQSALEAAEREKAEALSLN
          	**.   *::*       .    : .  :*  .*: *   *::: .        ::: **.

MG02781.1 	GSKTGTTGTS---------RYANARLGGN-TGSPMASQQNLRPYSQPAGKDTLAPQNNPL
NCU00675.1	GTN-GTHRNT---------VNNNNRLGVNGVTSPNGSNSNLRPSSSPTG-----PNGVQA
FG08819.1 	GSKTETTGTRGHLTVKTNGMNGNGLTNGHGPTSPYGSQYNLMRPHSSHG-HREKEREGTI
AN2496.1  	GSRTGTLPVR-------------GHAHANGTGSSISTNSPSTAHVATAG----------L
          	*:.: *                      :.  *. .::        . *           

MG02781.1 	AKLRKSSLRSN-ATPSPASS---GPIRSGVTSVDQLKMVLSGQVAPAVVRAQTSHGNGAG
NCU00675.1	GRTRKTSIRSNGGGGSPAHSTYRAKAQQPVTSVEQLKAVLSGHLQP----PPTSHG--IN
FG08819.1 	PRHRKGSVRSAHSPAALSTG-----NRGTVASVDQLKLVLSGNPPP------KTAG----
AN2496.1  	QTSRRMSMPDKTVTPNHNSS------RDSPVRVNELRRVLSVQAPDRDRRLSPLRG-RLD
          	   *: *: .         .      :   . *::*: *** :            *   .

MG02781.1 	GLGADDSTDSLVSYDMTVSEMSFNAGSVTYTPSHPVPRPPSRSRSHSRERRAASRSGAES
NCU00675.1	FQHSDDSDDSLVSYDMAPSELSFNP--AASGSRQGSPGNTSQAASSPPRRTSQDRTQQLK
FG08819.1 	LAGDDDSGDSMVSYDYSLSDMSFNP--ATQNDQPGSPTSVKRPGTSSKRGPLNTHP---P
AN2496.1  	ASNGSIISSSSESMVSGFSNSEFEGDGGSILLPTRDGQEPLDGDGMETPRPLSNGNNYGY
          	    .   .*  *     *: .*: .. :                               

MG02781.1 	PGGFDQLSRSNSFGKSNGNGRTIEDGRHDRHDDDGVPPVPPLPSGVSSPPLPPSRDGKSP
NCU00675.1	FGGPLVPGEESVVNGAGG-----QEGSNGAAGNLGVP-----ISRTTSRTQPQATTAHTT
FG08819.1 	LGGAPNLHEE-------------QNGDGSVPPVPPMPDMN-MLGGKRSPIQAMENSFQEK
AN2496.1  	LQTAFRASSSSIPPVPPIPHGISIPGGFPNDSQRSLPTPDRPSTASSRKQSLINGKFGIA
          	         ..    .         *         :*                       

MG02781.1 	SPRSPSTQAPVLPPVSLESSAIRPAKTRSLKSRGRHSRSGSIPSAPAPHAMRPATSAGHG
NCU00675.1	LPR-PSTSS-----------------KRSIKSR-AGSRAG------------PMSSSWLG
FG08819.1 	GRR------------------------RSLNSR---NGDG----------MRPKSVRSQE
AN2496.1  	APADDKTLHRQKSRTGVGLVNGAEVPEVAINGKSTGTHSYGLQEVEETSQRRDVQKLLEG
          	   . .:     .  .            :::.:.  .   .  .   .            

MG02781.1 	VVGGDSAFDLDALLLGIDTKDMQG--TLGNVTRPVY
NCU00675.1	EK--PPAMDLAALLKGIDSASISDIKSLGAGGKPPY
FG08819.1 	TK----TMDLQDLLRGIDSRPSEG--SLGNVTRPPY
AN2496.1  	VLS--SSEPASTLQPRVASNYSGRRSVTGGIGRPPY
          	  .  .:     *   : :      .  *   :* *
```
